# Improved Bioactivity of 3D Printed Porous Titanium Alloy Scaffold with Chitosan/Magnesium-Calcium Silicate Composite for Orthopaedic Applications

**DOI:** 10.3390/ma12020203

**Published:** 2019-01-09

**Authors:** Chun-Hao Tsai, Chih-Hung Hung, Che-Nan Kuo, Cheng-Yu Chen, Yu-Ning Peng, Ming-You Shie

**Affiliations:** 1 School of Medicine, China Medical University, Taichung 40447, Taiwan; ritsai8615@gmail.com (C.-H.T.); u102001403@cmu.edu.tw (Y.-N.P.); 2 Department of Orthopedics, China Medical University Hospital, Taichung 40447, Taiwan; cmuhd10227@gmail.com; 3 Department of Bioinformatics and Medical Engineering, Asia University, Taichung 40447, Taiwan; cnkuo@asia.edu.tw; 4 3D Printing Medical Research Institute, Asia University, Taichung 40447, Taiwan; 5 Institute of Oral Science, Chung Shan Medical University, Taichung 40447, Taiwan; chenyo8321@yahoo.com.tw; 6 3D Printing Medical Research Center, China Medical University Hospital, Taichung 40447, Taiwan; 7 School of Dentistry, China Medical University, Taichung 40447, Taiwan

**Keywords:** three-dimensional scaffolds, titanium, magnesium-calcium silicate, chitosan, osteogenesis, bone tissue engineering

## Abstract

Recently, cases of bone defects have been increasing incrementally. Thus, repair or replacement of bone defects is gradually becoming a huge problem for orthopaedic surgeons. Three-dimensional (3D) scaffolds have since emerged as a potential candidate for bone replacement, of which titanium (Ti) alloys are one of the most promising candidates among the metal alloys due to their low cytotoxicity and mechanical properties. However, bioactivity remains a problem for metal alloys, which can be enhanced using simple immersion techniques to coat bioactive compounds onto the surface of Ti–6Al–4V scaffolds. In our study, we fabricated magnesium-calcium silicate (Mg–CS) and chitosan (CH) compounds onto Ti–6Al–4V scaffolds. Characterization of these surface-modified scaffolds involved an assessment of physicochemical properties as well as mechanical testing. Adhesion, proliferation, and growth of human Wharton’s Jelly mesenchymal stem cells (WJMSCs) were assessed in vitro. In addition, the cell attachment morphology was examined using scanning electron microscopy to assess adhesion qualities. Osteogenic and mineralization assays were conducted to assess osteogenic expression. In conclusion, the Mg–CS/CH coated Ti–6Al–4V scaffolds were able to exhibit and retain pore sizes and their original morphologies and architectures, which significantly affected subsequent hard tissue regeneration. In addition, the surface was shown to be hydrophilic after modification and showed mechanical strength comparable to natural bone. Not only were our modified scaffolds able to match the mechanical properties of natural bone, it was also found that such modifications enhanced cellular behavior such as adhesion, proliferation, and differentiation, which led to enhanced osteogenesis and mineralization downstream. In vivo results indicated that Mg–CS/CH coated Ti–6Al–4V enhances the bone regeneration and ingrowth at the critical size bone defects of rabbits. These results indicated that the proposed Mg–CS/CH coated Ti–6Al–4V scaffolds exhibited a favorable, inducive micro-environment that could serve as a promising modification for future bone tissue engineering scaffolds.

## 1. Introduction

Repair or replacement of bone defects is still a problem for orthopaedic surgeons today, especially if there is a massive loss of bone mass due to trauma or disease. Therefore, within the past decade, bone scaffolds have grown in popularity, and demand for them has increased as an alternative for bone substitutes and as a replacement strategy for autografts and allografts. Proper selection of materials and fabrication methods is required in order to produce scaffolds with optimal osteoconductivity and osteoinductivity properties that are also bio-compatible and have sufficient mechanical properties to withstand frictional and compressional forces after implantation [1]. Furthermore, fabricated bone substitutes can reduce or prevent unwanted immunological responses and help avoid risk of infectious diseases such as HIV or hepatitis from donors [2]. Up until the present, various synthetic and biological materials have been available for the fabrication of bone scaffolds, but all of them have both limitations and advantages. Therefore, the search for a balanced material to fabricate an optimal bone scaffold is still on-going [3,4].

Metal implantable materials usually have both higher mechanical strength and ductility as compared to polymers or ceramic materials [5,6]. Therefore, metal materials are often considered as the first-choice candidates for bone tissue engineering. In fact, natural bone is actually a hierarchical composite characterized by a dispersion of inorganic apatite among organic fibers [7]. Of all the metals, Titanium (Ti) alloys are most frequently selected because they have a lower Young’s modulus as compared to their counterparts, stainless steel or CoCr. In addition to their ability to withstand specific forces, Ti alloys have been found to have a lack of cytotoxicity after implantations [8]. However, even though Ti alloys have one of the lowest Young’s modulus among the metal family, they still have mechanical properties that are higher than those characteristics of human bones, thus leading to stress shield effects and osteoporosis after implantations long-term. According to the Gibson and Ashby model, introduction of different types of porous structures onto scaffolds can aid in reducing the Young’s modulus thus leading to adjustable mechanical responses such as elastic limit, yield stress, etc. Porous structures allow better bone ingrowth and formation into the scaffolds, thus allowing better material-bone integration [5]. Unfortunately, the inherent bio-inertness of Ti alloys has led to limited improvements in hard tissue regeneration, which may significantly limit their applications in some orthopedic procedures, for example, in the repair of segmental bone defects [5]. However, traditional fabrication methods do not allow fabrication of complicated porous structures with high resolution, therefore it has not been easy to predict the mechanical properties of designed scaffolds beforehand [9]. It was only the recent development of additive manufacturing (AM) technology, especially the powder bed fusion method, that allowed us to fabricate complicated porous scaffolds for hard tissue engineering [10,11].

In addition, surface property is always considered to be a modifiable parameter that is critical to the success of scaffolds [12]. Numerous materials, such as bio-ceramics and calcium phosphate-based ceramics, have been used as coatings on the surface of Ti-based alloys scaffolds, and many reports have been made indicating that such coatings enhance the biological and physiological properties of Ti-based scaffolds [13]. Numerous studies have focused on calcium silicate (CS), especially in the area of hard tissue engineering because CS is reported to have higher levels of biocompatibility, biodegradability, and bioactivity as compared to other biomaterials [14]. It was found that both modification of CS with Ti elements and the incorporation of magnesium (Mg) with CS improves the chemical stability of CS scaffolds when immersed in simulated body fluid (SBF) [15]. In terms of biodegradation, CS was found to have a slower degradation rate as compared to other ceramics, and its by-products were reported to have anti-bacterial effects, thus making it suitable for application in bone tissue engineering [16]. A recent study by Chen et al. reported that Mg–CS has better degradation rates than neat CS, thus making Mg–CS more suitable for clinical applications [17]. In addition, CS was found to be hydrophilic in nature, and SBF immersed CS was reported to exhibit continuous release of Ca and Si ions throughout the entire immersion period [18,19]. These factors were found to enhance initial cellular adhesion, which led to subsequent downstream up-regulation of cell attachment, proliferation, differentiation, extracellular matrix secretion, and bone tissue formation [20]. Furthermore, Mg–CS ceramics were shown to have excellent bone-like hydroxyapatite precipitate abilities after SBF immersion. This apatite layer formation is a pre-requisite for materials to bond to bones [21].

As mentioned previously, bone is made up of inorganic apatite and organic fibers. This serves as the inspiration for us to attempt to design new inorganic-organic hybrid scaffolds for bone tissue engineering [22]. Chitosan (CH) is a natural positively charged polymer derived from the partial deacetylation of chitin and is a material widely used in biomedical applications because it is highly biocompatible, has antimicrobial effects, and is inexpensive [23]. Chitin can be found in the exoskeleton of crustaceans and some fungi. Furthermore, the by-products of chitosan are easily broken down by human enzymes such as lysozyme, therefore allowing it to be incorporated into glycoproteins for applications. Chitosan, in terms of composition and chemical structure, is akin to the extracellular matrix of bones and cartilages [24,25]. Therefore it is known to be flexible and highly ductile and thus is rarely used as a neat scaffold on its own [26]. Instead, several physicochemical properties of chitosan suggest that it is a suitable candidate for hybridization with other materials and subsequently used as a coating layer for metallic implants [27]. CH-based scaffolds have been found to have enhanced osteoconductivity, and upregulated bone regeneration has been observed in both in vivo and in vitro studies [28].

In this study, we used an Mg–CS/CH coating on 3D-printed Ti–6Al–4V scaffolds, which not only improved their surface bioactivity but also affected cell behavior. Therefore, our hypothesis is that Si ions may be released from Mg–CS/CH that have an effect on bone regeneration. We also conducted analyses of the coating morphology, physico-chemical properties, composition, and water contact angle. In addition, Human Wharton’s Jelly mesenchymal stem cells (WJMSC) were used in this study to analyze for scaffold efficacy by observing levels of cellular adhesion, proliferation, and osteogenesis when cultured on the developed Mg–CS/CH-coated Ti–6Al–4V scaffolds.

## 2. Materials and Methods

### 2.1. Porous Titanium Scaffolds Fabrication

The pre-alloyed Ti–6Al–4V powders (Ti64ELI) were purchased from Renishaw, England. The Ti–6Al–4V powder (Renishaw, Wharton Anderch, England) is specified in the ASTM F136 specification to ensure that the end product exhibits good corrosion resistance, good biocompatibility, low density, and excellent ductility if the process environment is well controlled. The particle size of the starting Ti–6Al–4V powders was about 15–45 µm. The additive manufacturing process was conducted using the Renishaw AM 400 system as the selective laser melting (SLM) equipment. Before the SLM process, the building chamber was vacuumed with a mechanical pump to 1 × 10^−2^ torr and then filled with Ar to 1 atm to ensure that the oxygen content was low enough to avoid serious oxidation during the SLM process. Using the computer aided design software (123D Design Ver.2.2, Autodesk, San Rafael, CA, USA), we fabricated the cylindrical porous Ti–6Al–4V scaffold (10 mm diameter × 10 mm height) using the laser to melt the selected region on the powder bed layer by layer, where the max laser powder was approximately 400 W; the spot size was approximately 70 µm, and the layer thickness was approximately 30 µm. After the SLM process, all of the samples were heat treated in a vacuum annealing furnace to eliminate thermal stress that was generated during the high cooling rate SLM process.

### 2.2. Mg–CS Powder Preparation

Mg–CS powder was prepared in accordance to previously reported methodologies [17]. Briefly, reagent grade CaO (Sigma-Aldrich, St. Louis, MO, USA), SiO_2_ (High Pure Chemicals, Saitama, Japan) and MgO (Sigma-Aldrich) powders were mixed and used as basal materials. CaO, SiO_2_ and MgO were mixed to a composition of 65:25:10, respectively. The mixture was then placed into a high temperature furnace set at 1400 °C for a duration of 2 h for sintering, after which the MG–CS powder was placed in 99.5% ethanol and ball milled (S100, Retsch, Hann, Germany) for 6 h, followed by another 6 h of drying at 100 °C. The 10 g powder was then placed into 100 mL of 0.1 N acetic acid and stirred for 12 h followed by triple rinsing with deionized water. Subsequently, the suspensions underwent filtration through 0.22 μm filter paper and were then dried in a 60 °C oven.

### 2.3. Mg–CS/CH Coating

0.5 g of chitosan (CH) powder was dissolved in 100 mL 0.1 N acetic acid and stirred for 12 h at room temperature. The Mg–CS concentrations used in this study were 0%, 0.2%, and 0.5% (referred to as CS0, CS20, and CS50, respectively). In addition, 500 μL of Mg–CS/CH solution was dropped onto Ti–6Al–4V scaffold in a 48-well plate for 1 h and then transferred into a freezer at −80 °C for 12 h and freeze-dried. Afterwards, the scaffolds were submerged in 0.1 M NaOH for 30 min followed by triple rinsing with distilled water and dried subsequently in a 40 °C oven.

### 2.4. Characterization

First, the phase composition of the Mg–CS/CH-coated Ti–6Al–4V scaffolds was analyzed using X-ray diffractometry (XRD; Bruker D8 SSS, Karlsruhe, Germany) with pre-set settings of 30 kV and 30 mA and a scanning speed of 1°/min. In order to evaluate the hydrophilicity of the composites, we also analyzed the contact angle of each scaffold at 37 °C. In brief, a drop of 20 μL ddH_2_O was placed on the surface of the Mg–CS/CH-coated Ti–6Al–4V scaffold, and the images were taken with a camera after 10 min. The water contact angle was analyzed using ImageJ (National Institutes of Health, ver.1.40). In addition, an EZ-Test machine (Shimadzu, Kyoto, Japan) was used to measure the compressive strengths of the scaffolds, with a pre-set loading rate of 2.5 mm/min. The stress–strain curves of the Mg–CS/CH-coated Ti–6Al–4V scaffolds were analyzed, and the load-deflection values were recorded as the maximal compressive force that the scaffolds could withstand. Results were obtained from ten independent tests, and data were recorded as mean ± SD. A scanning electron microscope (SEM; JSM-7800F, JEOL, Tokyo, Japan) accelerating voltage 3 kV was used to observe the morphology of the Ti–6Al–4V coated scaffolds.

### 2.5. Ion Release

The Mg–CS/CH-coated Ti–6Al–4V scaffolds were immersed in SBF solution at 37 °C for various time periods. The SBF solution was made up of 7.9949 g of NaCl, 0.2235 g of KCl, 0.147 g of K_2_HPO_4_, 0.3528 g of NaHCO_3_, 0.071 of g Na_2_SO_4_, 0.2775 g of CaCl_2_, and 0.305 g of MgCl_2_·6H_2_O in 1000 mL of distilled H_2_O. The solution was made to be similar to human blood plasma. Hydrochloric acid and trishydroxymethyl aminomethane (Tris) were used to adjust the solution to pH 7.4. After immersion for various periods of time, the released Ca and Si ion concentrations were analyzed using an inductively coupled plasma-atomic emission spectrometer (ICP-AES; Perkin-Elmer OPT 1MA 3000DV, Shelton, CT, USA). Results were obtained from six independent tests, and data were recorded as mean ± SD. 

### 2.6. Extracellular Matrix Secretion and Adsorption Analysis

Prior to in vivo testing, all the Mg–CS/CH-coated Ti–6Al–4V scaffolds were placed into 75% ethanol and exposed to 20 min of UV light for sterilization. Human Wharton’s Jelly mesenchymal stem cells (WJMSCs) were obtained from the Bioresource Collection and Research Center (BCRC, Hsin-Chu, Taiwan) and were subsequently grown in a commercial mesenchymal stem cell medium (#7501, Sciencell, Carlsbad, CA, USA) to passage 4–8. Cells at a density of 5 × 10^4^ were directly seeded onto the scaffolds in a 48-well plate with culture medium and incubated in an incubator pre-set at 5% CO_2_ atmosphere at 37 °C. After 1 h and 3 h of culture, an enzyme-linked immunosorbent assay kit (Invitrogen, Waltham, MA, USA) was used to analyze the level of Col I and FN secretion at the various time points. This was done in accordance to the manufacturer’s instructions and determined using a standard curve. The results were obtained in triplicate from five independent experimental analyzes were performed for each group.

After being cultured for 3 h, the amount of Col I and FN secreted from WJMSC onto the scaffold surface was quantified using the enzyme-linked immunosorbent assay [29]. The cells were detached using a trypsin-EDTA solution (Cassion) after being washed 3 times with cold PBS. Scaffolds were then washed 3 times with PBS containing 0.1% Tween 20 (PBS-T; Sigma-Aldrich) followed by blocking with 5% bovine serum albumin (Gibco, Waltham, MA, USA) in PBS-T for 1 h. Dilutions of primary antibodies were set at 1:500. After this procedure, the scaffolds were incubated with anti-human FN and anti-human Col I antibody (GeneTex, San Antonio, TX, USA) for 3 h at room temperature. Afterward, scaffolds were washed 3 times with PBS-T for 5 min and incubated with horseradish peroxidase–conjugated secondary antibodies for 1 h at room temperature with shaking. The scaffolds were then washed 3 times with PBS-T for 10 min each, and then One-Step Ultra TMB substrate (Invitrogen) was added to the wells and developed for 30 min at room temperature in the dark; after this, an equal volume of 2 M H_2_SO_4_ was added to stop and stabilize the oxidation reaction. The colored products were then transferred to new 96-well plates and read using a Tecan Infinite 200^®^ PRO microplate reader (Tecan, Männedorf, Switzerland) at 450 nm with reference at 620 nm according to the manufacturer’s recommendations. All experiments were performed in triplicate. Additionally, the scaffolds with cells which were followed the above procedure and incubated with β-actin antibodies were used as a control.

### 2.7. Cell Morphology

For this test, the cells were grown in a mesenchymal stem cell medium (Sciencell) up to passage 3–6. After culture with WJMSC (10^4^ cell/per scaffold) for 6 h and 1 day, the Mg–CS/CH-coated Ti–6Al–4V scaffolds were removed from the medium and washed twice with PBS. In order to fix the WJMSC, all specimens were immersed in a 2% glutaraldehyde (Sigma-Aldrich) solution at room temperature for 2 h. The specimens were subsequently dehydrated by being subjected to sequential dehydration for 20 min with ethanol (50%, 60%, 70%, 80%, 90%, 95%, and 99.5%). After drying and coating with gold, the morphology of the WJMSC on the different Mg–CS/CH-coated Ti–6Al–4V scaffolds was examined using SEM.

### 2.8. Cell Proliferation

WJMSCs at 10^4^ cells per well were seeded onto the scaffolds placed in a 48-well plate filled with cultured medium and incubated in an incubator pre-set at 5% CO_2_ atmosphere at a temperature of 37 °C. The specimens were cultured for various time periods of 1, 3, and 7 days. After every time point, a PrestoBlue^®^ (Invitrogen, Grand Island, NY, USA) assay was used to analyze for the level of cell viability. Briefly, the medium was removed, and the wells were rinsed twice with cold PBS. PrestoBlue^®^ solution was mixed with fresh medium to a ratio of 1:9 and was placed into each well. Subsequently, the specimens were incubated for an additional 60 min, after which the solution was transferred to a 96-well plate, and a Tecan Infinite 200^®^ PRO microplate reader was used to measure for the optical density (OD) with a pre-set setting of 570 nm and a 600 nm reference wavelength. Results were obtained in triplicate from six independent tests and recorded.

### 2.9. Osteogenesis Assay

Osteogenesis was done on cells cultured on scaffolds with commercially available differentiation kits (StemPro™ osteogenesis differentiation kit, Gibco) for 3 and 7 days. An alkaline phosphatase (ALP) assay kit (Sigma, St. Louis, MO, USA) was used to measure for the level of alkaline phosphatase according to the manufacturer’s instructions. The ALP activity was calculated according to the standard curve using ELISA under 405 nm. In addition, the osteocalcin (OC) protein concentration was measured using an OC enzyme-linked immunosorbent assay kit (Invitrogen). This test was done according to the manufacturer’s instructions. The OC concentration was calculated with reference to the standard curve. Blank cartridges were used as controls. Results were obtained in triplicate from six independent tests and recorded.

### 2.10. Alizarin Red S Stain

The levels of calcium secretion and deposition by WJMSCs were measured using Alizarin Red S staining (Sigma-Aldrich). For the test, cells were cultured on scaffolds in an osteogenic differentiation kit for 3 weeks, and the staining was done in accordance with previously reported procedures [16]. Briefly, 4% paraformaldehyde (Sigma-Aldrich) was used to fixate the specimens by placing them in the solution for 15 min, after which the specimens were placed in 0.5% Alizarin Red S with a pH of 4.0 for 15 min at room temperature. The BX53 Olympus microscope (Olympus, Tokyo, Japan) was used to capture images of cells at 100× magnification. The Alizarin Red S was also quantified using a solution of 20% methanol and 10% acetic acid in water. After 15 min, the liquid was transferred to a 96-well, and the quantity of Alizarin Red was determined using a spectrophotometer at 450 nm.

### 2.11. Scaffold Implantation into Femur Defects of Rabbits

All in vivo experimental protocols and statement to confirm that all methods were carried out in accordance with relevant guidelines and regulations, and all experimental protocols were approved by the Ethical Committee for Animal Experiments of China Medical University, Taichung, Taiwan. 6.5 × 10 mm specimens from the Ti group and CS50 group were implanted into femoral bone defects of adult New Zealand rabbits 12 rabbits with defects created both for right and left posterior limbs. The rabbits were positioned in a stereotaxic frame and immobilized during surgery. The hair over the femur of the animals was shaved, and the underlying skin was aseptically prepared using iodine scrub. The underlying periosteum was accurately incised and elevated to obtain sufficient exposure for the trephine. A trephine with an outer diameter of 6.5 mm was used to remove bone and create critical size defects in the femur 6.5 mm in diameter and 10 mm in depth. The scaffolds from the various groups in this study were then implanted at the site of the 6.5-mm critical lesions. Lastly, the periosteum and the subcutaneous tissue were closed sequentially with sutures. The animals were kept on a surgical bed until they woke and had free access to food and water thereafter. Six weeks post-implantation, the rabbits were sacrificed by CO_2_ asphyxiation, and the femur bone specimens of the rabbits were harvested and fixed in 10% formalin for 48 h, after which they were rinsed with PBS several times, embedded in OCT (KMA-0100-00A, CellPath Ltd., Newtown, UK), and sectioned (10-mm thick). Subsequently, 5 μm longitudinal sections were prepared per specimen using a microtome sawing technique. The sections were prepared and stained using a Von Kossa kit (ScyTek, Logan, UT, USA), according to the manufacturer’s instructions. Von Kossa staining in red was used to observe the difference between the osteoid tissue and the calcified bone. The sections were examined using the BX53 Olympus fluorescence microscope (Olympus, Tokyo, Japan) at 200× magnification. Finally, a histological analysis was performed, and the area of newly-formed bone was quantified using the image analysis system.

### 2.12. Statistical Analyses

Significant differences between the means were measured using a one-way analysis of variance. Scheffé’s multiple comparison test was used to determine for significance of the deviations in each data. For all tests, results with a *p* value < 0.05 were considered statistically significant.

## 3. Results and Discussion

### 3.1. The Characterization of CS/CH-Contained Ti Scaffold

**Figure 1:**
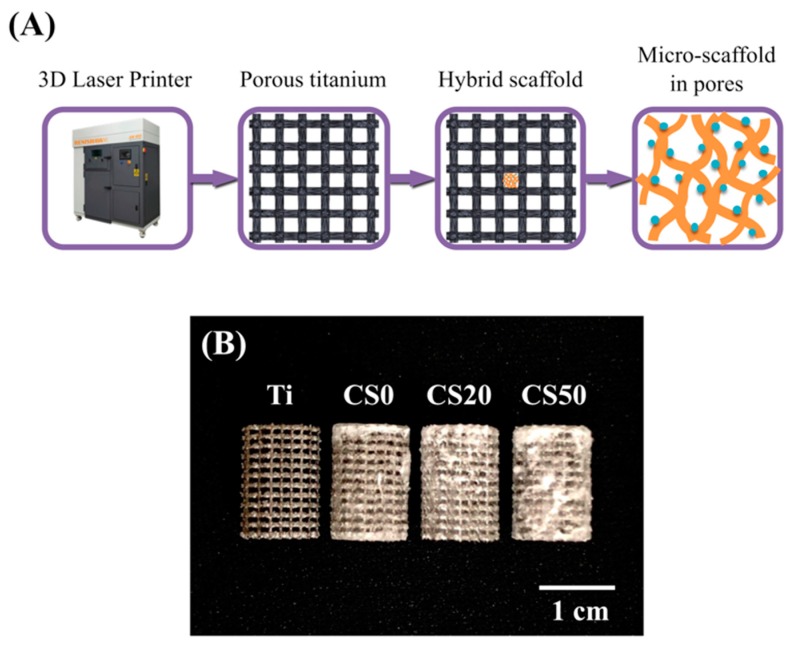
(**A**) Scheme showing the fabrication of the 3D printed Ti–6Al–4V scaffold and bioactivation by an Mg–CS/CH coating; (**B**) Photographic image of the Mg–CS/CH-coated Ti6–Al–4V scaffold.

**Figure 2:**
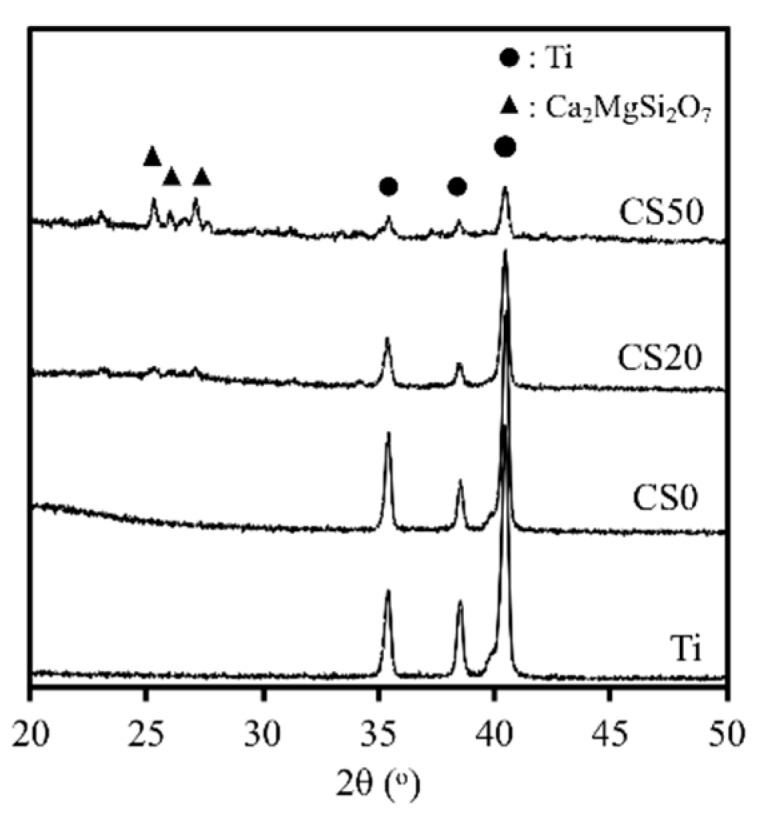
XRD patterns of Mg–CS/CH-coated Ti–6Al–4V scaffold, where the markers indicate the Ti and Ca_2_MgSi_2_O_7_.

A 3D-printed Ti–6Al–4V scaffold coated with Mg–CS/CH was fabricated (Figure 1A). The composite scaffolds had well connected and defined pores, morphology, and architecture (Figure 1B). Figure 2 shows the XRD patterns of the Mg–CS/CH-coated Ti–6Al–4V scaffolds. A lack of CS-related peaks was noted in pure Ti and CS0. With increases in Mg–CS concentration, a gradual increase in the main characteristic peaks could be seen. However, there was only a weak peak of the akermanite phase for specimens up to the CS20 groups [30]. The characteristic peaks only became distinct at CS50.

**Figure 3:**
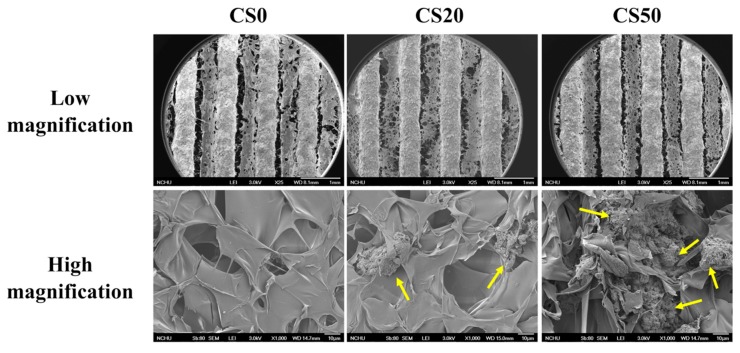
Low magnification and high magnification SEM images of the Mg–CS/CH-coated Ti–6Al–4V scaffold surface. The yellow arrows indicate calcium silicate powder aggregate.

The surface morphology of the Mg–CS/CH-coated Ti–6Al–4V scaffolds, at both low and high magnification are shown in Figure 3. Macro-pores with proper inter-connected structures were clearly visible on the Ti–6Al–4V scaffolds while distinct linear architectures with obvious Mg–CS/CH substrates between each line were clearly visible on the remainder of the Mg–CS/CH coated scaffolds (low magnification). As seen from the SEM images, there was no observable mineral on the CS0 specimens. These results of the SEM were concurred with the XRD analysis. On the other hand, increases in Mg–CS concentration led to an increase in mineral aggregates and density (yellow arrow). Pore sizes have been reported to play a huge role in influencing the formation of new bone and related regeneration processes such as the vascularization and in-growth of bone tissues [4]. In brief, a minimum value of 100 μm for pore sizes has been recommended for effective nutrients and oxygen transport to ensure cell viability and maintenance of basic cell functions [4]. In addition, it has been suggested that inter-connected pore sizes be kept within the range of 200–350 μm for effective osteogenesis and vascularization. In our study, our 3D-printed Ti–6Al–4V scaffold had a pore size of about 350 μm, which is well within the parameters suggested by other scholars. Based on the high magnification of the CS0 SEM micrographs, there were traces of chitosan coating found among the structures. Similar to reports made by others, this non-uniform, fissured coating was commonly observed if the hybrid coating solution contains <30% chitosan. However, for CS20 and CS50, increased homogeneity of the hybrid Mg–CS/CH coating was observed, and when in conjunction with increased concentrations of Mg–CS and CS50, a more uniform morphology was observed as compared to the rest of the groups. This surface roughness might be an advantage for enhancing cellular behavior. Surface properties have been reported to enhance osteoblast activity and improve cellular behaviors such as adhesion, differentiation, and proliferation [31].

**Figure 4:**
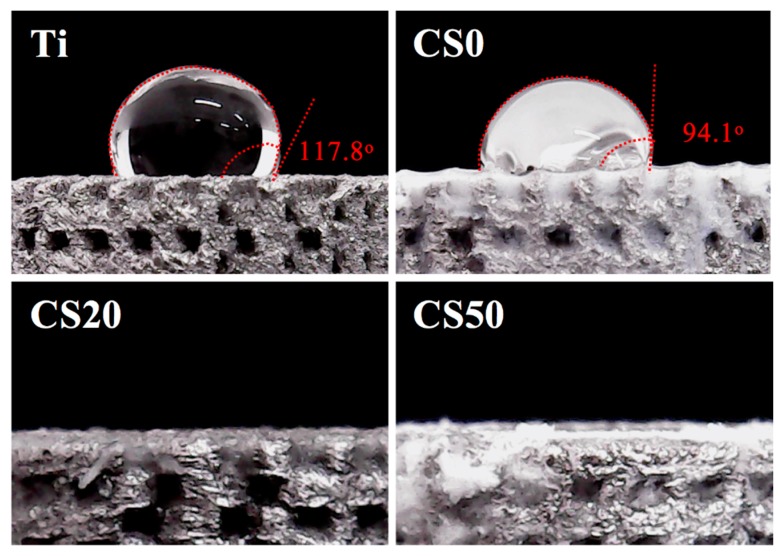
Water contact angle of different various Mg–CS/CH-coated Ti–6Al–4V scaffolds.

Next, the hydrophilic behavior of pure Ti and Mg–CS/CH-coated Ti–6Al–4V scaffolds were considered with reference to the contact angle, as shown in Figure 4. The high contact angle (>90°) of the Ti–6Al–4V and CS0 scaffolds were examined and compared to the CS20 and CS50 groups. There was no significant effect found on the hydrophilicity of the pure Ti scaffold with the CH coating. The contact angle of the Mg–CS/CH-coated Ti–6Al–4V scaffolds could be decreased from 94.1°, 0°, and 0° with increases in the CS coating concentration using CS0, CS20, and CS50, respectively. It was also found that a water contact angle of less than 75° enhanced cellular behavior. A higher Mg–CS content, led to higher hydrophilic behavior in the scaffolds. The results indicated that the CS0 scaffold is hydrophobic, that the material coated with Mg–CS/CH composite was hydrophilic, and that the material with the best hydrophilicity was favorable for cell behavior [32]. With the assumption that the water contact angle influences cellular behavior, we posited that the Mg–CS coated scaffolds developed in the present study were able to positive influence cellular behavior because they are hydrophilic as compared to the hydrophobic Ti–6Al–4V scaffolds.

**Figure 5:**
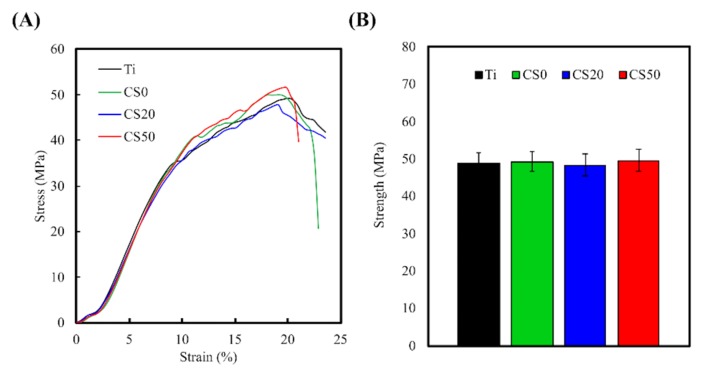
(**A**) The strain-stress curves and (**B**) compressive strength of different Mg–CS/CH-coated Ti–6Al–4V scaffolds (*n* = 10).

Representative stress–strain curves of Mg–CS/CH-coated Ti–6Al–4V scaffolds at same strain rate of 0.04 s^−1^ are exhibited in Figure 5. All the Mg–CS/CH-coated Ti–6Al–4V scaffolds were analyzed at least three times until mechanical fracture occurred. The Young’s modulus of the CH with various amounts of Mg–CS content (CS0, CS20, and CS50) was close to that of the Ti–6Al–4V scaffold (approximately 0.65 GPa) (Figure 5A). In addition, the maximum compressive strengths of the 3D-printed Ti–6Al–4V, CS0, CS20, and CS50 scaffolds were 49.3 ± 0.9, 49.7 ± 1.7, 48.5 ± 1.4 MPa, and 50.3 ± 1.6 MPa, respectively (Figure 5B). However, traditional titanium alloys have a significantly higher Young’s modulus as compared to natural bone [6]. The excessive Young’s modulus causes a stress shielding effect that indicates sustained loading energy between a metal implant and natural bone tissue [33]. As a result, solid metal implants can easily cause osteoporosis in this area. Therefore, porous structures are usually fabricated into the new designed biomedical implants to reduce the Young’s modulus to match that of natural bones [34].

### 3.2. Ion Release

**Figure 6:**
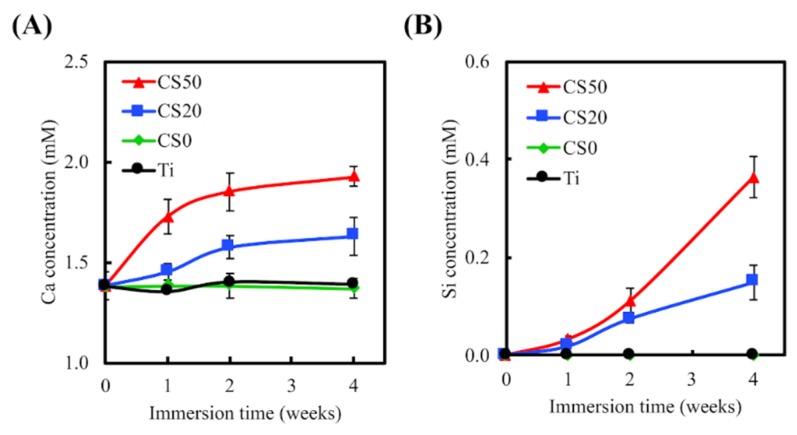
(**A**) Ca and (**B**) Si ion concentrations of simulated body fluid (SBF) following Mg–CS/CH-coating of the Ti–6Al–4V scaffolds immersed for different time periods (*n* = 6).

The ICP-AES analysis indicated a minimal release of Ca and Si ions from the Ti–6Al–4V alloys and CS0 after immersion in SBF for various time points, as shown in in Figure 6. On the other hand, the concentrations of Ca and Si ions increased significantly in the CS20 and CS50 groups after 4 weeks of immersion. After 4 weeks of immersion, the Ca ion concentrations for both CS20 and CS50 were increased to 1.63 ± 0.10 mM and 1.93 ± 0.05 mM (an increase of approximately 18% and 40%), respectively, whereas the Si ion concentrations for both CS20 and CS50 were increased to 0.15 ± 0.04 mM and 0.36 ± 0.04 mM, respectively. These results further support the increased Mg–CS content discussed in the results above. Previous studies have indicated the calcium concentrations do not influence the proliferation of osteoblasts in the short term, but do change the cell morphology during long cell culture periods [35]. Thus, it was verified that the local Ca concentration in the environment was the factor contributing to the modulation of cell behavior, including cell proliferation and differentiation [36]. Moreover, the Ca ion plays a pivotal role in regulating the interactions between cellular materials or cell matrices that vary cell functions during culturing [37]. Furthermore, there has been evidence found indicating that presence of Si ions enhances the proliferation of bone cells, as well as increasing collagen production, ALP activities, and osteocalcin levels [38]. Furthermore, low levels of Ca and Si ions have been shown to promote osteoblast and fibroblast proliferation [39,40]. Shie et al. demonstrated that an Si concentration of 1 mM in a cell culture medium enhanced the proliferation of osteoblast-like cells [41].

### 3.3. Extracellular Matrix Secretion

**Figure 7:**
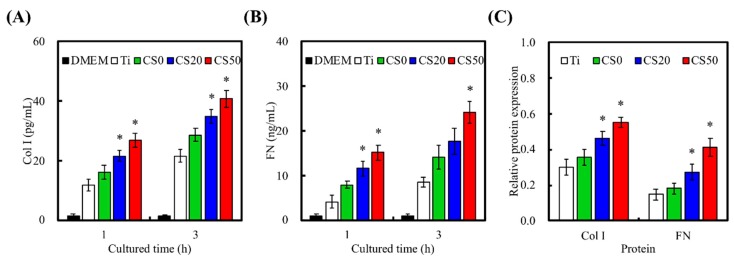
(**A**) Col I; (**B**) FN secretion from Wharton’s Jelly mesenchymal stem cells (WJMSCs) and (**C**) adsorption on the Mg–CS/CH-coated Ti–6Al–4V scaffold surface (*n* = 5). “*” indicates a significant difference (*p* < 0.05) compared to CS0.

Following initial adhesion onto the Mg–CS/CH-coated Ti–6Al–4V scaffolds, cells secrete components of extracellular matrix (ECM), which further promotes cellular attachment and influences subsequent cell function. Adult stem cells are involved in various tissue regeneration processes throughout a person’s life [42]. However, the major bottlenecks in the use of adult stem cells are their highly invasive harvesting procedures and their proliferation and differentiation behavior. However, there have been several success studies reported in the literature for isolation and characterization of stem cells from Wharton’s jelly tissue [43]. Wharton’s jelly mesenchymal stem cells (WJMSCs) are thought to constitute potential candidates for tissue regeneration, and driving them into the osteogenic lineage shows great promise for new bone regeneration [38]. Therefore, to determine the ability of the Mg–CS/CH-coated Ti–6Al–4V scaffold proposed in this study to support initial cellular adhesion, we studied the level of Col I (Figure 7A) and FN (Figure 7B) secreted after cells were seeded for 1 and 3 h. At both time periods, cells seeded on the CS20 and CS50 scaffolds exhibited significantly higher levels (*p* < 0.05) of Col I secretion of 35.0 ± 2.4 and 40.8 ± 2.7 pg/mL, respectively, as compared to only 28.6 ± 2.2 and 21.6 ± 2.3 pg/mL for the CS0 and Ti–6Al–4V scaffolds, respectively. However, in terms of FN secretion, only the CS50 scaffolds exhibited significantly higher levels (*p* < 0.05) after 1 and 3 h of culture. The CS20 scaffolds were only able to induce a significant difference in FN secretion at the 1 h mark (*p* > 0.05). FN secretion in the CS50 scaffolds was approximately 300% that of the Ti–6Al–4V scaffolds and 158% of the CS0 scaffolds at 24.1 ± 2.5 ng/mL after 3 h of culture. The effect of substrates on the adsorption of Col I and FN were also examined. As shown in Figure 7C, there was no statistically significant difference (*p* > 0.05) in Col I and FN adsorption between Ti and CS0. The value for CS20 and CS50 groups were significantly (*p* < 0.05) higher CS0. In a previous study, Col and FN were the main proteins found to be expressed and secreted throughout the phases of cell adhesion [38]. In addition, the adsorption of ECM proteins was found to positively influence in vivo biomaterial live bone bonding [44]. Col I and FN were found to contain numerous types of cellular binding substrates, such as Arginylglycylaspartic acid sequences, which bind to various components or receptors of cell membranes or live tissues to enhance downstream effects. Putting it simply, Col I and FN are adsorbed onto the surface of materials and thus basically act as a biological platform for further cellular attachment. As such, it has been reported that different quantities of ECM protein present on surfaces heavily determines the levels of cellular adhesion, which in turn influences cellular behavior [45]. Our study showed that hybrid CS–CH coated Ti–6Al–4V scaffolds can be used as a potential surface modification technique and that they have the ability to enhance the expression of Col I and FN proteins due to improved initial cell adhesion with the surface of the Ti–6Al–4V scaffolds. Furthermore, as mentioned above, increased release of Si ions by the materials presented herein may also enhance Col I or FN secretion.

### 3.4. Cell Adhesion

**Figure 8:**
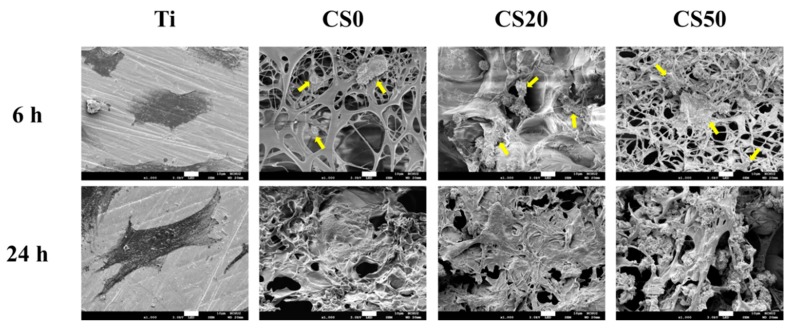
SEM images of WJMSCs adhered on the Mg–CS/CH-coated Ti–6Al–4V scaffold surface for 6 h and 24 h. The yellow arrow refers to WJMSC. The scale bar is 10 µm.

The cross-talk between surface composition and stem cell and subsequent bone-forming osteoblastic cells is of great importance, but remains unclear. Therefore, SEM micrographs of cellular adhesion on the Mg–CS/CH-coated Ti–6Al–4V scaffolds after 6 and 24 h of culture are shown in Figure 8. As shown in Figure 8, the SEM images indicate that the majority of the WJMSCs cultured on the CS0 surface remained spherical in shape and exhibited poor spreading behavior, whereas a higher level of cell spreading into a typically polygonal morphology could be observed on CS50 after seeding for 6 h. It is worth noting that a confluence of the WJMSC monolayer was formed on the Mg–CS/CH-coated Ti–6Al–4V scaffold after 24 h of seeding. On the other hand, our results proved that CS50 released more Si ion to stimulate WJMSC secreted Col I and FN that were able to promote cell adhesion in the short term. There have been several studies indicating the formation of anchors between adhesion receptors and suggesting that their ligands can trigger the formation of focal adhesion to further regulate proliferation and differentiation processes through activating a series of signaling pathways [44]. Therefore, we reasoned that although the difference in cell attachment was observed only in the short-term, it should affect the protein expression of the subsequent osteogenesis differentiation pathway.

### 3.5. Cell Proliferation

**Figure 9:**
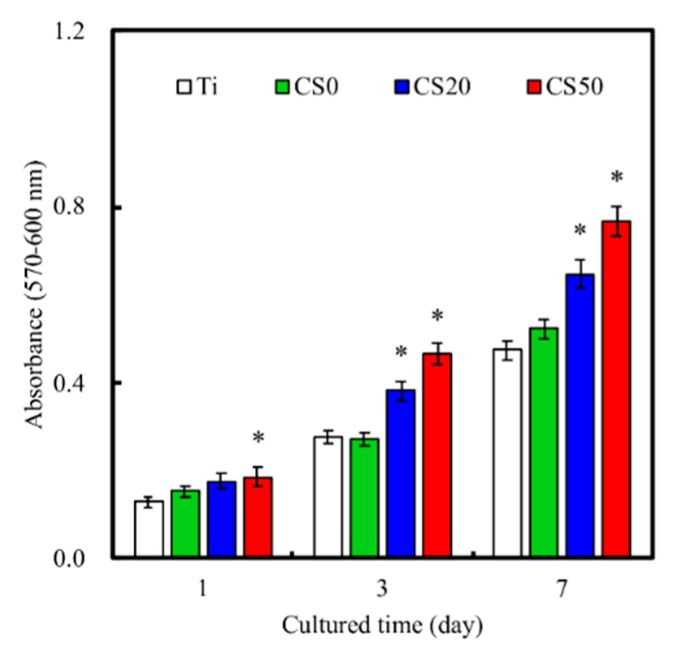
The proliferation of WJMSC cultured on the Mg–CS/CH-coated Ti–6Al–4V scaffold surface for different time periods (*n* = 6). “*” indicates a significant difference (*p* < 0.05) compared to CS0.

The quantitative analysis results of cellular proliferation are provided in Figure 9. It can be seen that the CS50 scaffolds enhanced cellular proliferation to a significantly higher degree (*p* < 0.05) for all time periods and that the CS20 scaffolds had significantly higher (*p* < 0.05) proliferation from day 3 onwards. Therefore, it is worth noting that proliferation was enhanced based on the concentration of Mg–CS. There was a gradual increase in cell proliferation for all groups from days 1 to 7, and there were no significant differences (*p* > 0.05) between Ti–6Al–4V and CS0. The absorbance values of cells cultured on CS0, CS20, and CS50 for 1 day were 1.2, 1.3, and 1.4 times higher than that on Ti, respectively. In addition, the proliferation rate (day 7/day 1 ratio) of CS0, CS20, and CS50 were 3.4, 3.7, and 4.2, respectively. In general, these data implied that the Mg–CS/CH coating contributed to the fabrication of functional Ti–6Al–4V scaffolds that were shown to be cell friendly and allowed the uniform adhesion of cells that would subsequently up-regulate downstream cellular activities. In previous reports, the results proved that Ca and Si play important roles in metabolism, collagen secretion, and hard tissue mineralization [21,46]. These ionic releases from scaffolds could be responsible for the up-regulation of cell proliferation [47,48]. Previous studies have shown that 0.17 to 2.51 mM of released Si ion concentrations enhance cellular functions [49], and this result was similar to reports made by Wu et al. [50]. In this context, we hypothesized that certain concentrations of Si ions release from the Mg–CS/CH-coated Ti–6Al–4V scaffolds are able to guide WJMSCs and upregulate cellular behavior [51].

### 3.6. Osteogenic Differentiation, Mineralization, and Bone Formation

**Figure 10:**
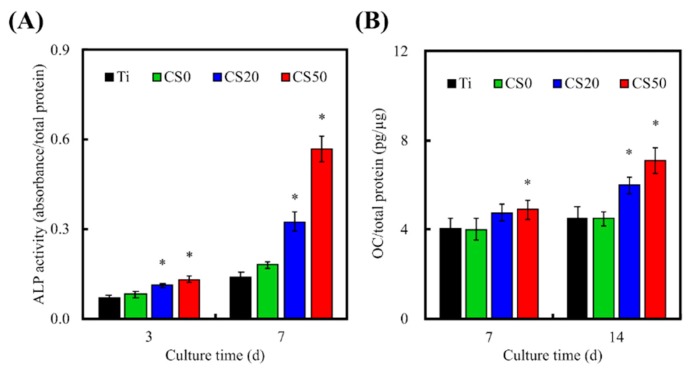
(**A**) Alkaline phosphatase (ALP) and (**B**) OC protein expression in the WJMSCs cultured on the Mg–CS/CH-coated Ti–6Al–4V scaffold surface for 3 and 7 days (*n* = 6). “*” indicates a significant difference (*p* < 0.05) compared to the specimen without CS0.

Levels of osteogenic differentiation can be used to determine whether there will be potential subsequent successful bone formation. Many enzymes and genes are involved in different stages of osteogenic differentiation and bone formation, of which alkaline phosphatase (ALP) is a protein enzyme that is commonly secreted in the early phases of bone mineralization and thus is generally used as a marker to analyze the differentiation status of cells. In this study, levels of ALP expression in cells cultured for 3 and 7 days were evaluated (Figure 10). Cells seeded on the CS20 and CS50 scaffolds secreted significantly higher amounts (*p* < 0.05) of ALP as compared to those seeded on the CS0 and Ti–6Al–4V scaffolds after 3 days of culture. On the other hand, after 7 days of culture, the cells seeded on CS0, CS20, and CS50 scaffolds secreted significantly higher (*p* < 0.05) amounts of ALP as compared to the control Ti–6Al–4V scaffolds. However, there were no significant differences (*p* > 0.05) between CS20 and CS50 for all time periods. ALP was observed to increase gradually over time as well as increase with increasing concentrations of Mg–CS. These data indicated that the Mg–CS/CH coated Ti–6Al–4V scaffolds enhanced the secretion of osteogenic protein ALP, thus implying that these scaffolds were able to promote osteogenesis in cells, thus leading to subsequent improved bone formation. Similarity, a significant synergistic interaction was observed between CH and Mg–CS in enhancing osteogenic differentiation of WJMSC (*p* < 0.05), as well as increasing OC secretion, as shown in Figure 10B. From our previous study, it was shown that CS-based materials enhance cellular adhesion due to the presence of integrins, which work by activating osteogenic-related signaling pathways by up-regulating the expression of focal adhesion kinase (FAK) and MAPE/ERK [41]. Thus, CS50 might cause an increase in ECM adsorption and bio-mineralization, thus enhancing osteogenic differentiation [44]. Therefore, our ALP secretion profile is consistent and in agreement with the cellular adhesion, ECM protein adsorption, and proliferation profiles, as indicated above. In addition, Zhai et al. proved that the Si ion released from a Ca–Si–Sr bioceramic significantly affects the OC gene and protein expression that not only activates Smad1/5-BMP2 signaling pathways, but also promotes osteogenic gene transcription in conjunction with the transcription factor Runx2 [18].

**Figure 11:**
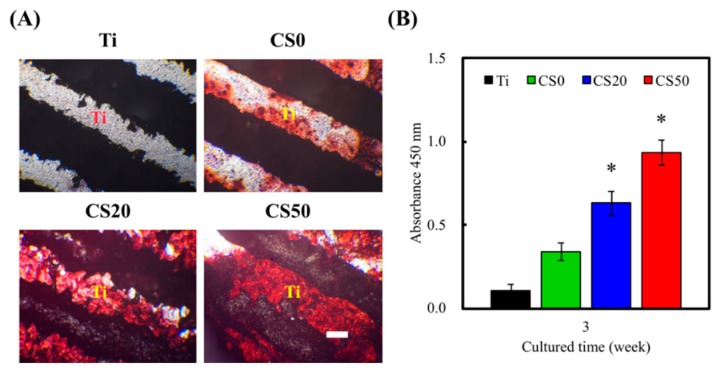
(**A**) Alizarin Red S staining and (**B**) quantification of calcium nodules deposited on the on the Mg–CS/CH-coated Ti–6Al–4V scaffold surface cultured with/without WJMSC for 3 weeks.

**Figure 12:**
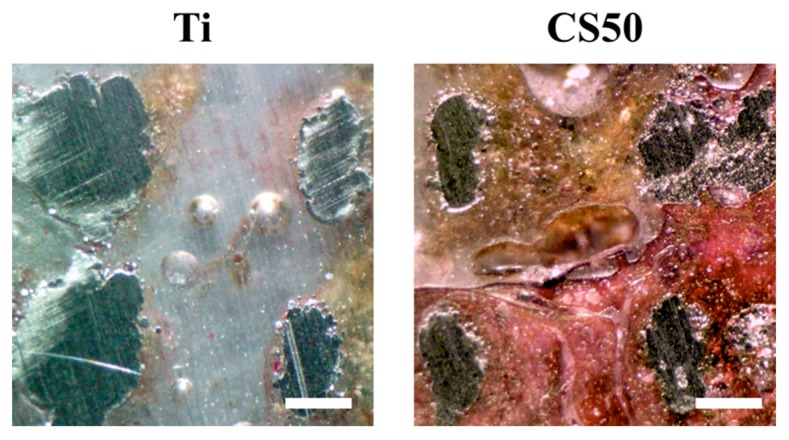
Evaluation of in vivo bone formation after implantation by means of Von Kossa staining. Ti and CS50 scaffolds after being implanted for 4 weeks. Scale bar: 200 μm.

Alizarin Red S was used to evaluate the calcium deposition capability of cells so as to better understand the influence of CS–CH coatings on osteogenic mineralization in cells. As shown in Figure 11A, the color of Alizarin Red S stains changed from light pink to darker shades of pink when the concentration of CS was increased, thus indicating that there were increased amounts of calcium mineral deposits found on the scaffolds. It is worth noting that the above observation was made on both the neat scaffolds and on the cell-seeded scaffolds. Therefore, this showed that neat Mg–CS/CH scaffolds had a baseline of calcium deposits that increased gradually with Mg–CS concentrations. In addition, the amounts in CS20 and CS50 groups were significantly higher (*p* < 0.05) than the amounts in CS0 group (Figure 11B). The above results explain that CS20 and CS50 groups were more beneficial to mineralization of the WJMSCs. In the in vivo test, the Van Kossa staining results showed there was less bone regeneration in the Ti scaffold after 6 weeks. Based on this contrast, it could be seen that the new bone regeneration in the defects filled with the CS50 group was rather limited and was mostly located around the macropores (Figure 12). Under a histomorphometric assay, a higher percentage of new bone area was examined in the CS50 groups (14.27% ± 1.18%) as compared to the Ti groups (2.54% ± 0.84%). These results show that the osteogenic differentiation on the CS20 and CS50 scaffolds was higher and that the cells exhibited better spreading behavior as compared with the Ti scaffolds without Si ion content, and the ALP of the WJMSCs on both the CS30 and CS50 scaffolds was also significantly higher than that on the Ti and CS0 scaffolds [52]. However, an overdose of Si ion causes a negative effect on stem cells, and a Si concentration that is too low may result in a lack of bio-function. Numerous reports have shown Si involvement in bone formation, mineralization, and regeneration [53]. For the cell seed scaffolds, there was a larger area of Alizarin Red S positive staining, and a denser stain was observed for the CS50 and CS20 scaffolds as compared to the rest of the specimens. Consistent with the above findings, our results showed that the Mg–CS/CH coated scaffolds exerted a positive influence on osteogenic differentiation in the cells and thus enhanced the calcium deposits on the scaffolds. The results from the Mg–CS/CH coated Ti–6Al–4V scaffolds further affirmed our previous reported findings that CS-based scaffolds are potential suitable candidates for future applications in bone tissue engineering.

## 4. Conclusions

In this work, it was demonstrated that Mg–CS/CH can be successfully coated onto Ti–6Al–4V scaffolds with simple immersion techniques to overcome the limitations of neat Ti–6Al–4V scaffolds. Pore sizes, morphologies, and architectures that are critical to subsequent hard tissue regeneration were maintained after coating, thus showing that such modifications do not alter or modify the physical properties of Ti–6Al–4V scaffolds. Our results demonstrated that such modifications can render Ti–6Al–4V surfaces hydrophilic, which is critical for cellular adhesion and proliferation. Our scaffolds can provide suitable micro-environments to support cellular adhesion, proliferation, and differentiation. Osteogenic differentiation and mineralization were enhanced, which makes such coatings a potential candidate for future regenerative medicine applications. Our results provide a platform for future innovative alternatives that can be used to create more robust scaffolds with enhanced biological behavior.
